# Expression of the scaffold connector enhancer of kinase suppressor of Ras 1 (CNKSR1) is correlated with clinical outcome in pancreatic cancer

**DOI:** 10.1186/s12885-017-3481-4

**Published:** 2017-07-21

**Authors:** Humair S. Quadri, Taylor J. Aiken, Michael Allgaeuer, Radim Moravec, Sean Altekruse, S. Perwez Hussain, Markku M. Miettinen, Stephen M. Hewitt, Udo Rudloff

**Affiliations:** 1 Thoracic and Gastrointestinal Oncology Branch, Gastrointestinal Oncology Section, Investigator Center for Cancer Research, National Cancer Institute, Building 10 - Hatfield CRC, Room 4-5950, Bethesda, MD 20892 USA; 2 Laboratory of Pathology, National Cancer Institute, Bethesda, MD USA; 3 Surveillance Informatics Branch, National Cancer Institute, Bethesda, MD USA; 4 Laboratory of Human Carcinogenesis, National Cancer Institute, Bethesda, MD USA; 5 Experimental Pathology Laboratory, Laboratory of Pathology, National Cancer Institute, Bethesda, MD USA

**Keywords:** Pancreas cancer, Biomarker, Correlative tissue study, Scaffold connector enhancer of kinase suppressor of Ras 1 (CNKSR1)

## Abstract

**Background:**

Despite the near universal occurrence of activating codon 12 KRAS somatic variants in pancreatic cancer, there is considerable heterogeneity in the molecular make-up, MAPK/ERK pathway activation states, and clinical outcome in this disease. We analyzed the expression levels of CNKSR1, a scaffold that influences MAPK/ERK pathway activity, in clinical pancreas cancer specimens and their impact on survival of patients with pancreatic cancer.

**Methods:**

Immunohistochemical staining for CNKSR1 expression was performed on 120 specimens from three independent pancreatic cancer tissue registries, phospho-ERK levels were measured in 86 samples. Expression was divided into CNKSR1 low and CNKSR1 high and correlated with clinicopathological variables including overall survival using multivariate Cox proportional hazard ratio models.

**Results:**

CNKSR1 expression was increased in tumors compared to matched normal uninvolved resection specimens (*p* = 0.004). 28.3% (34/120) of patient specimens stained as CNKSR1 low compared to 71.7% (86/120) of specimens which stained as CNKSR1 high. High CNKSR1 expression was more prevalent in low grade tumors (*p* = 0.04). In multivariate analysis, low CNKSR1 expression status was independently correlated with decreased overall survival (HR = 2.146; 95% CI 1.34 to 3.43). When stratifying primary, non-metastatic tumor biopsies by CNKSR1 expression, resection was associated with improved survival in patients with high CNKSR1 expression (*p* < 0.0001) but not low CNKSR1 expression (*p* = 0.3666). Pancreatic tumors with nuclear in addition to cytoplasmic CNKSR1 staining (32/107) showed increased nuclear phospho-ERK expression compared to tumor with cytoplasmic CNKSR1 staining only (*p* = 0.017).

**Conclusion:**

CNKSR1 expression is increased in pancreatic tissue specimens and was found to be an independent prognostic marker of overall survival. CNKSR1 may help to identify patient subgroups with unfavorable tumor biology in order to improve risk stratification and treatment selection. Cellular distribution of CNKSR1 was correlated with nuclear pERK expression.

## Background

While advances in the understanding of cancer biology, screening and risk-reducing interventions, and improved treatments have significantly reduced cancer mortality overall, pancreatic cancer remains a deadly disease. The American Cancer Society estimates 53,070 new cases and 41,780 deaths from pancreatic cancer in the United States during 2016 and predicts that pancreatic cancer will rank second of all cancer-related mortalities by the year 2030 [1, 2]. Neither current chemotherapy nor molecular therapy provides patients with an extension of survival measured by more than a few months, or the hope for sustained tumor regressions. Even in the minority of patients who are able to undergo surgical resection, median overall survival remains poor [3]. To date, only a few biomarkers have been associated with survival outcomes in pancreatic cancer [3, 4]. Considering the variability of clinical outcome and the uncertainty of the role of surgical resection in cancers at high risk for early progression, prognostic biomarkers accurately stratifying patients for individualized clinical decision-making would fill an unmet clinical need.

Activating somatic KRAS mutations are nearly omnipresent and a hallmark in the genetic make-up of pancreatic ductal adenocarcinoma (PDAC) [5]. While KRAS mutations themselves have been associated as prognostic markers, there is considerable and significant heterogeneity in the activation states of the downstream MAPK/ERK pathway, the molecular landscape, response to therapy, and clinical outcome across pancreatic cancers [6–9]. The MAPK/ERK (MEK) pathway downstream of RAS has been the topic of significant research efforts in attempting to target and inhibit the oncogenic progression of RAS-mutant tumors. Certain scaffolding kinase proteins are essential to the spatiotemporal regulation of MAPK/ERK pathway signaling, as well as for regulating and integrating input from and output to other key signaling pathways involved in cellular homeostasis [10, 11]. The Kinase Suppressor of Ras-1 (KSR1) is a well-examined scaffolding protein; it has been shown to mediate tumor progression in pancreas cancer and may govern response to treatment [12, 13]. For example, KSR1 can compete with binding partners of BRAF and directly modulate response to small molecule inhibition of the MAPK pathway at the RAF level and it has been shown to be dysregulated in endometrial and colon cancers [14–16]. CNKSR1 (connector enhancer of the Kinase Suppressor of Ras-1), a regulator and binding partner of KSR1, is another scaffolding protein which is less understood. Its role in pancreatic cancer biology, or as a biomarker, remains to be explored.

Current data suggests that CNKSR1 has multiple roles cancer biology, with some reports demonstrating that CNKSR1 interacts with tumor suppressors and others describe its scaffolding protein interactions as oncogenic [17, 18]. These include, for example, connecting Ephrin B stimulation via small GTPases to c-Jun N-terminal kinase (JNK) activation resulting in net cancer cell migration, or promoting cancer cell proliferation through the Akt-FoxO signalling axis [19, 20]. Recent studies using phosphomimetic mutants of CNKSR1 have identified phosphorylation sites in the scaffold critical for nuclear translocation and activation of MAPK pathway genes [21]. However, to date all CNKSR1 analysis in the context of pancreatic cancer has been performed at a molecular level with no translational or clinically oriented application. Using pancreatic tumor tissues from three independent cohorts, we aimed to evaluate the expression levels of CNKSR1 and its association with clinicopathological parameters and survival in pancreatic cancer. In addition we aimed to assess the association of CNKSR1 expression levels with MAPK pathway activity as measured by phospho-ERK. The observed association of CNKSR1 expression and survival outcome suggests scaffolding proteins of the RAS-MAPK pathway may account, in part, for the observed heterogeneity of PDAC biology, and clinically may aid in improved future patient stratification.

## Methods

### Study participants and tissue microarray (TMA) composition

De-identified cancer tissues included in this analysis were confirmed to be pancreatic ductal adenocarcinomas based on pathology slide review at the National Cancer Institute. The analytic dataset included 120 cases, including 99 from the Iowa, Hawaii and Los Angeles Surveillance, Epidemiology, and End Results (SEER) Residual Tumor Registries pancreatic cancer tissue microarray (TMA) [22]. Another 18 cases were patients treated at the University of Maryland at Baltimore Hospital (Baltimore, MD) and three more patients who were treated at the Clinical Center of the National Institutes of Health (Bethesda, MD). Appropriate ethical and transfer of material approvals were obtained from originating sites, as well as the NCI. A commercially purchased TMA of 71 cases (U.S. BioMax, Inc., Rockeville, MD) and a PDAC TMA from 47 patients treated at Stony Brook University (Stony Brook, NY) were used as a secondary cohort to confirm similar CNKSR1 expression distributions.

### Case attributes

The analytic dataset included demographic and clinical data, enabling analyses of CNKSR1 expression by age, race, gender, grade, resection status, the TNM variables lymph node status (N0; No regional lymph node metastasis, N1; Regional lymph node metastasis, and NX; Regional lymph nodes cannot be assessed) and distant metastasis (M0; No distant metastasis, and M1; Distant metastasis), SEER stage (localized, regional, and distant), radiation, and primary tumor location. Grade of tumor differentiation was determined upon initial diagnostic workup by the primary pathologist and derived from original pathology reports and data available within the SEER tumor registry. Histologic grade was based on overall extent of glandular differentiation within the resected specimen and, with the limitations of reviewing small tissue cores on a TMA, was re-confirmed in select cases. No re-classifications of the original grading upon re-review were made. Of the different grading systems the WHO 2010 [WHO Classification] classification was used defining Grade 1 as well differentiated (>95% of tumor composed of glands), Grade 2 moderately differentiated (50% - 95% glands), and grade 3 poorly differentiated (< 50% glands) [23]. Person months from diagnosis to date of last follow-up or death were recorded. Time contributed by patients that were alive at last follow-up and those that died of causes other than pancreatic cancer was censored as ending in a non-event. All cases with missing information were included in proportional hazard ratio calculations after performing a sensitivity analysis which showed negligible effects of excluding missing data.

### Immunohistochemistry

Immunohistochemical staining for CNKSR1 (mouse monoclonal antibody CNKSR1 (clone 46), Santa Cruz Biotechnology, TX, USA, #sc-135,870; dilution 1:200) was performed on a Leica BOND-MAX autostainer (Leica Microsystems, IL, USA). Antigen retrieval was for 25 min with Bond Epitope Retrieval Solution 2 (Leica Biosystems Newcastle, UK, #AR9640). Primary antibody was incubated for 30 min at room temperature. For detection the BondMax avidin biotin free polymer-based detection system (Bond Polymer Refine Detection #DS9800) was used with diaminobenzidine as chromogen.

CNKSR1 coordinates signal transduction through interaction with proteins of distinct pathways in the cytoplasm [21]. Despite a fraction of tumors also showing concomitant expression of CNKSR1 in the nucleus, only cytoplasmic staining was scored for clinical correlative studies since if adopted as a prognostic test it would be more feasible to interpret only one parameter. Across all cases, staining for CNKSR1 was very uniform within each single tumor sample. Therefore, a simplified approach of scoring CNKSR1 immunohistochemistry was applied evaluating only staining intensities and not proportions of tumor cells stained [24, 25]. CNKSR1 expression was evaluated based on intensity semiquantitatively on a four-tier scale (0 = negative, 1 = weak/background, 2 = moderate/positive, 3 = strongly positive). CNKSR1 shows minimal expression in lymphoid tissues according to RNA-Seq data and immunohistochemical staining from the Human Protein Atlas (Human Protein Atlas available from www.proteinatlas.org) [26]. Samples of lymph nodes and intratumoral lymphocytes were therefore used as negative controls.

Immunostaining for p-ERK1/2 was developed using a rabbit monoclonal antibody (Cell Signaling, Cat#4376) at 1:200 dilution. Staining was performed on the SEER Pancreas Cancer TMA only using standard IHC described above. With phosphorylation of extracellular-signal-regulated kinase (ERK) inducing nuclear translocation staining was predominantly nuclear with a few cases also showing cytoplasmic staining. Scoring of p-ERK1/2 was done blinded to the CNKSR1 results using standard intensity scores above (0 = no staining, 1 = weak staining, 2 = moderate staining, 3 = strong staining). In addition, the percentage of p-ERK1/2 positive cells within an examined tumor core was scored and recorded as well.

**Fig. 1:**
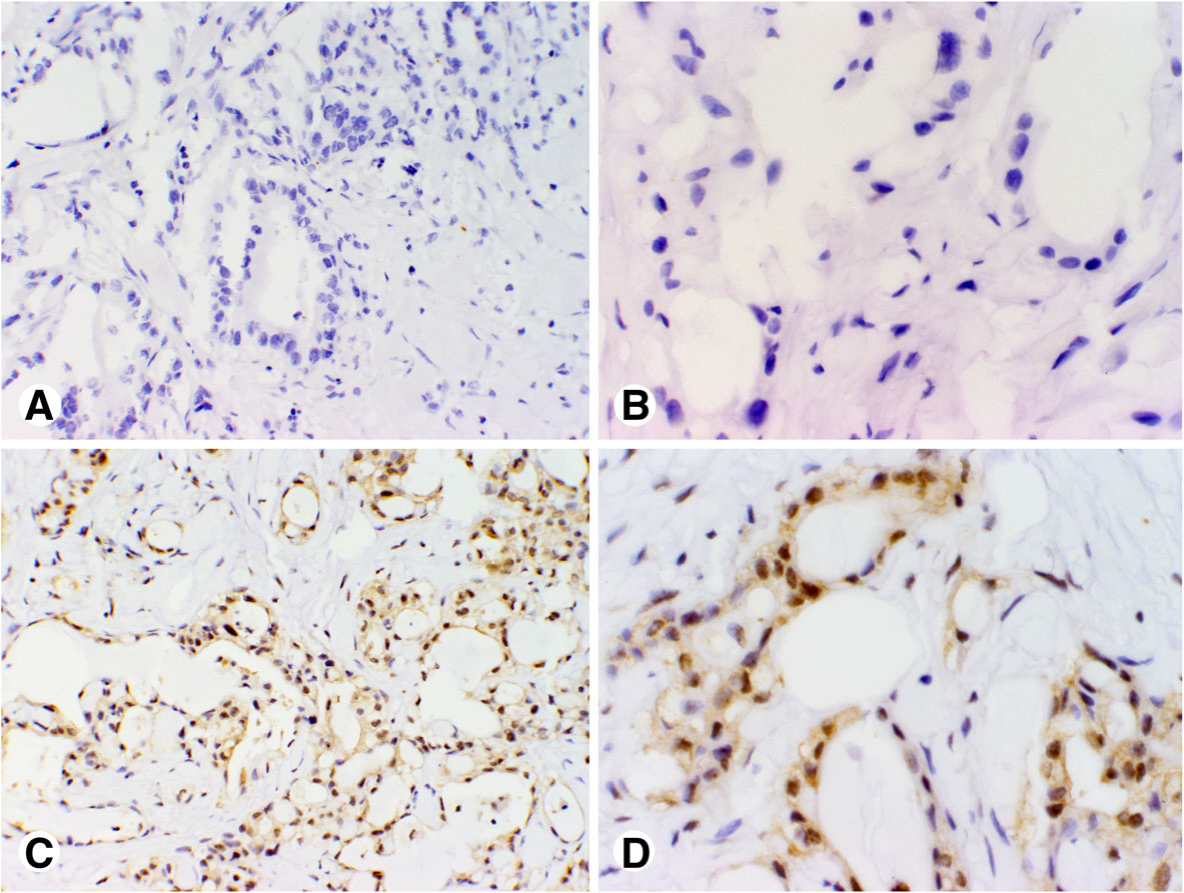
Representative photomicrographs of pancreatic cancer tissue microarray (TMA) cores illustrating intensities of CNKSR1 immunohistochemical staining scored as low: **a**, **b** no staining for CNKSR1 (score 0); **c**, **d** weak CNKSR1 (score 1+) staining; only cytoplasmic staining was scored. Magnification: **a**, **c** × 200; **b**, **d** × 400

**Fig. 2:**
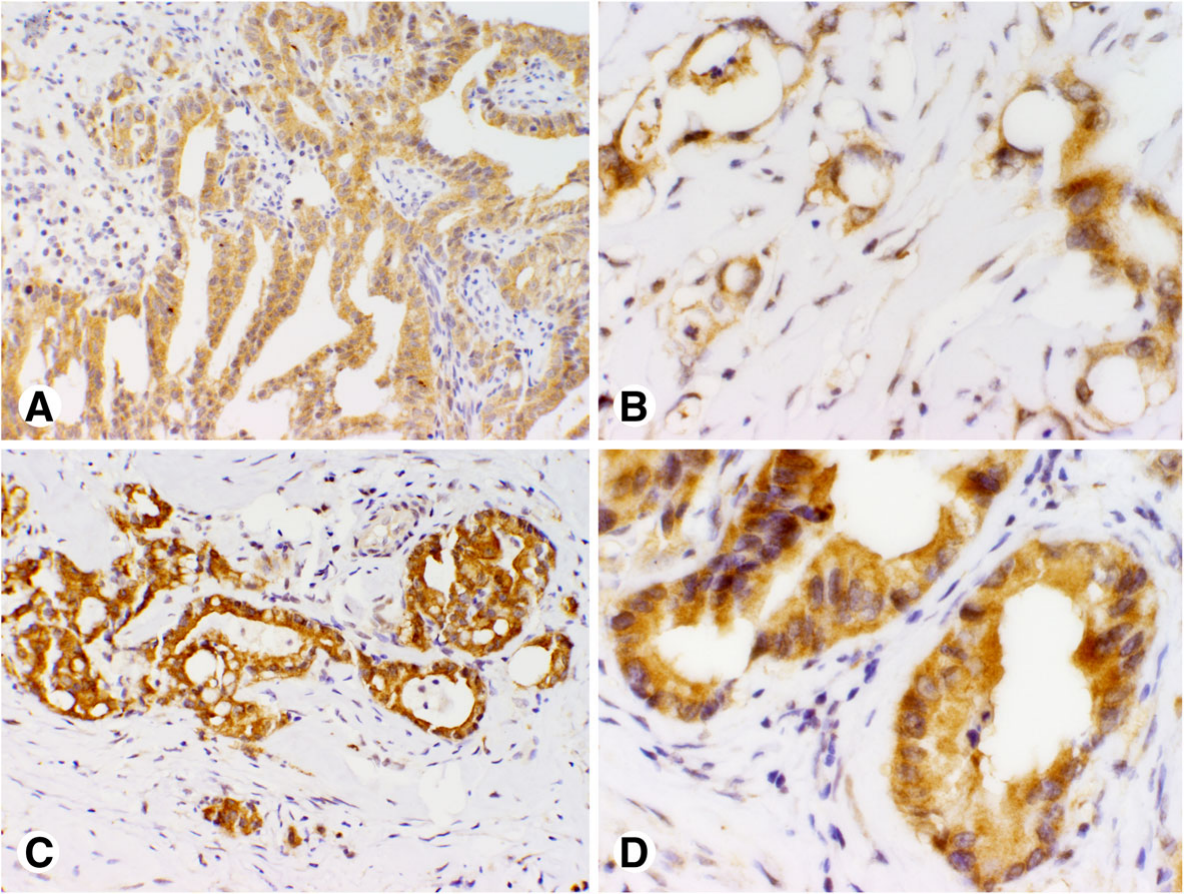
Photomicrographs of pancreatic cancer tissue microarray (TMA) cores of high CNKSR1 immunohistochemical staining: **a**, **b** positive for CNKSR1 (score 2+); **c**, **d** strongly positive for CNKSR1 (score 3+). Magnification: **a**, **c** × 200; **b**, **d** × 400

**Fig. 3:**
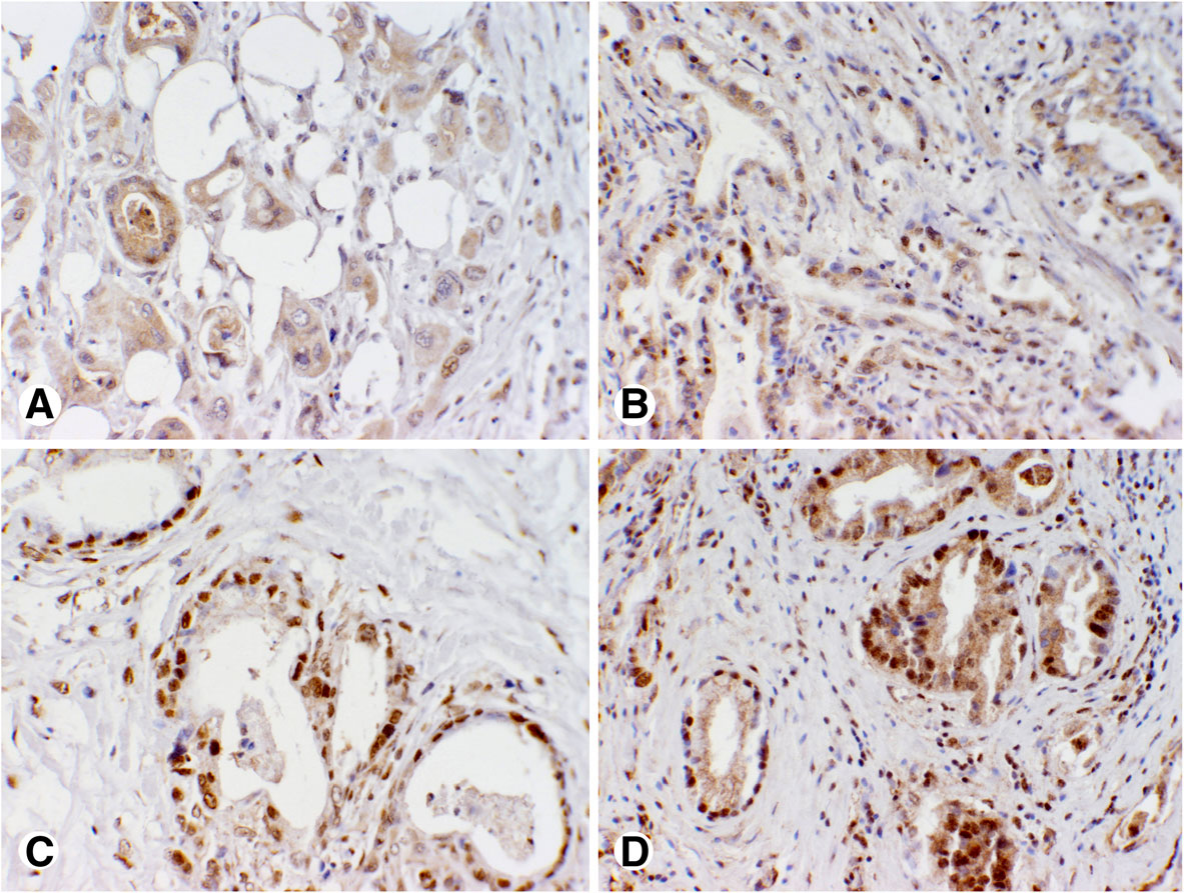
Photomicrographs of pancreatic cancer tissue microarray (TMA) cores of p-ERK immunohistochemical staining: **a** no staining for p-ERK (score 0); **b** weak p-ERK (score 1+) staining; **c** moderate p-ERK (score 2+) staining; **d** strong p-ERK (score 3+) staining. Magnification: **a**-**d**: ×200

Representative CNKSR1 staining patterns scored based on intensity of immunostaining in PDAC tissues are shown in Figs. 1 and 2. Representative p-ERK staining patterns scored based on intensity of immunostaining (no staining for p-ERK (score 0); weak p-ERK (score 1+), moderate p-ERK (score 2+), and strong p-ERK (score 3+) staining) in Fig. 3. Staining intensities were grouped as dichotomous variables, defining scores 0–1 as low and 2–3 as high expression levels [25]. Evaluation of staining was carried out independently by two pathologists (MM and MA) blinded to patients’ outcome and pathological stage. Discrepant scores were discussed to reach an agreement.

### Statistical analysis

Matched tumor and normal pancreatic tissues were compared using Wilcoxon matched-pairs signed rank test. Correlation of CNKSR1 and phospho-ERK expression levels was assessed by the Pearson’s correlation coefficient test (r; 2-tailed). Nuclear p-ERK expression levels in tumors were compared with the Mann Whitney U test (2-tailed) with cellular distribution of CNKSR1 (cytoplasmic only vs cytoplasmic and nuclear). Product-limit survival estimates were plotted using the Kaplan-Meier method with significance determined by log-rank test (PROC LIFETEST, SAS v 9.4, Cary, NC). Multivariate analysis was performed using Cox regression proportional hazard models (PROC PHREG, SAS v 9.4, Cary, NC) to estimate the risk of death among subjects with high CNKSR1 expression (reference group) compared to those with low CNKSR1 expression. A final model was developed using a stepwise variable selection process to adjust for gender, age, stage, grade, race, resection and radiation. Sensitivity analyses were performed after excluding cases with missing information on SEER stage (2), TNM stage (49) grade (1), resection (1) and radiation (4).

## Results

### CNKSR1 is overexpressed in human pancreas cancer

**Fig. 4:**
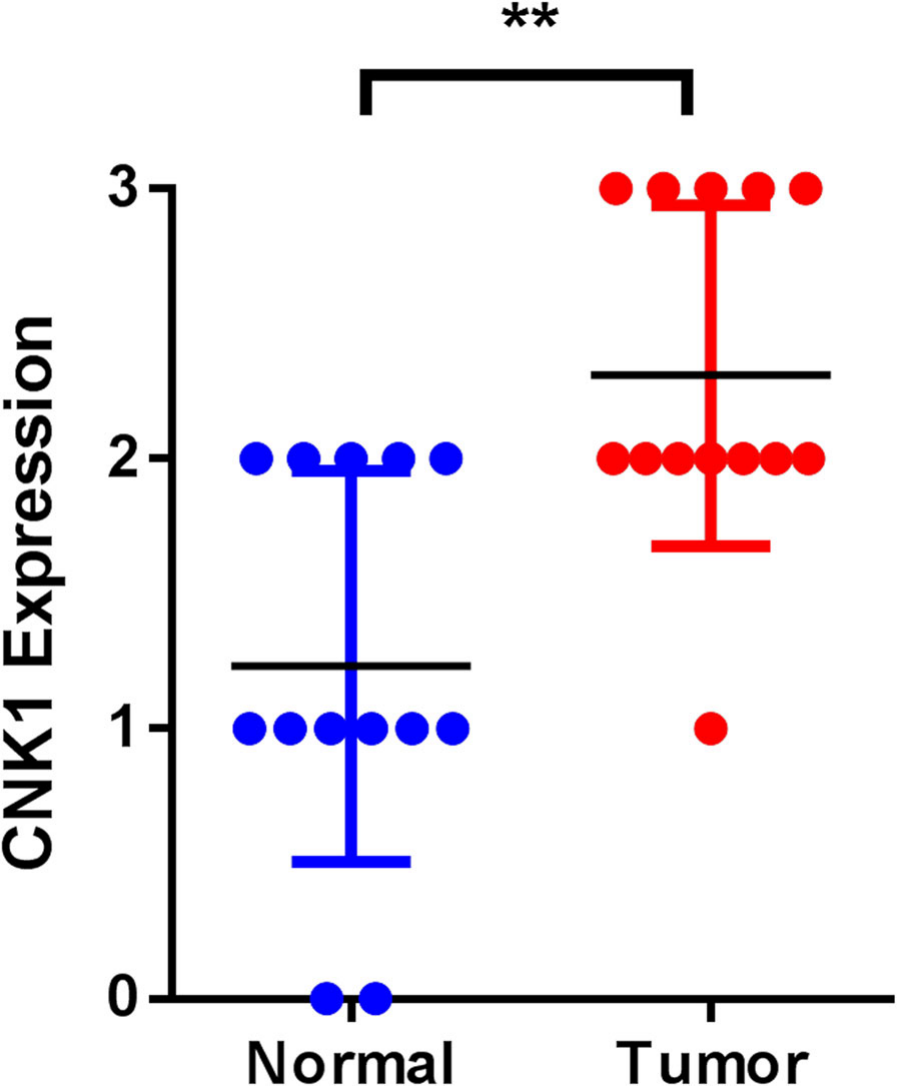
CNKSR1 expression in matched tumor and normal pancreatic tissues. Increased CNKSR1 expression levels were observed in tumor tissues compared to matched normal tissue by Wilcoxon matched-pairs signed rank test (** *p* = 0.004)

To examine if CNKSR1 expression is dysregulated in pancreas cancer we first compared CNKSR1 expression measured by intensity of immunostaining in 13 randomly chosen matched tumor and normal pancreatic tissues from the SEER Pancreatic Cancer TMA. Figure 4 shows elevated CNKSR1 protein expression levels in pancreatic tumors compared to matched, uninvolved controls (*p* = 0.004).

### CNKSR1 expression levels are heterogeneous in pancreatic adenocarcinoma

**Fig. 5:**
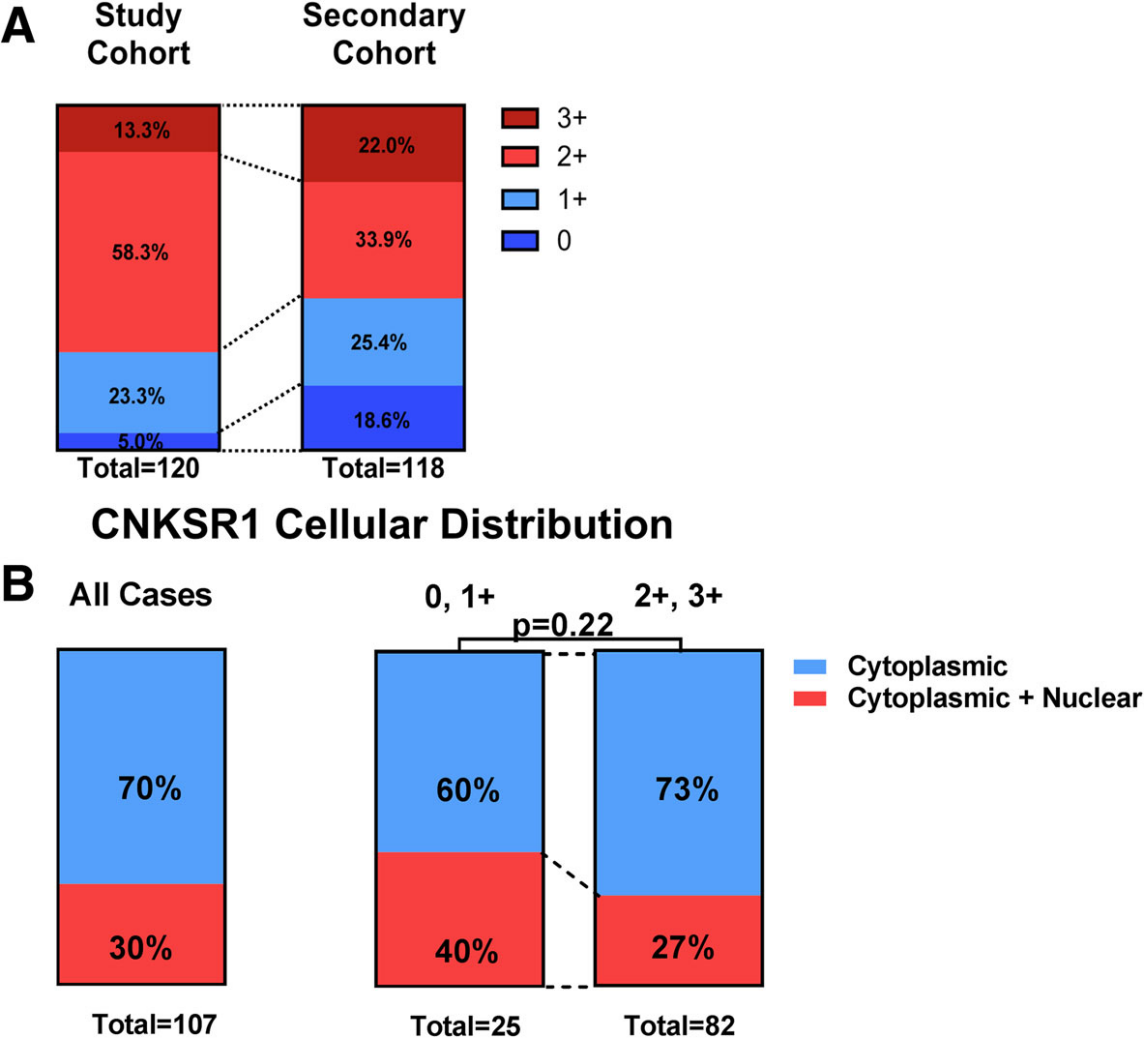
**a** Comparison of CNKSR1 expression of study cohort and secondary validation cohort. **b** Cellular distribution pattern of CNKSR1 showed primarily cytoplasmic expression in pancreatic cancer specimens. Nuclear staining of CNKSR1 was not associated with cytoplasmic CNKSR1 expression levels (0, 1+ vs 2+, 3+; *p* = 0.22; chi square test, 2-tailed)

Combining cases from all three cohorts, 6 cases showed no expression (5.0%), 28 cases were scored as 1+ (23.3%), 70 cases as 2+ (58.3%), and 16 cases as 3+ (13.3%) (Fig. 5a). To validate the expression pattern used for the clinical outcome associations in an independent cohort, 71 cases from a commercially available pancreatic cancer TMA and 47 cases from a TMA constructed at an outside institution were subject to the same CNKSR1 staining and reviewed by the same study pathologists (MM, MA). CNKSR1 low (0 and 1+ expression) versus CNKSR1 high (2+, 3+) comprised 28.3 and 71.7% of cases in the study cohort and 44.1 and 55.9% of cases in the validation cohort suggesting similar expression patterns across the different arrays. In the study cohort 30% of cases also showed some degree of nuclear staining (Fig. 5b). Nuclear staining was lower than cytoplasmic expression levels. There was no association between cytoplasmic CNKSR1 expression levels (0, 1+ vs 2+, 3+) and presence of nuclear CNKSR1 staining (*p* = 0.22) (Fig. 5b).

### CNKSR1 expression is prognostic of clinical outcome in pancreas cancer

Table 1 presents demographic and clinical characteristics of the pancreatic cancer patient specimens used for clinical outcome associations (SEER, UMD, and NIH). Table 2 presents the demographics and tumor characteristics by CNKSR1 expression status. The majority of cases were 65 years of age or older at the time of diagnosis (59%), regardless of CNKSR1 expression status. The gender distribution of cases was relatively balanced, including by CNKSR1 expression status. A higher proportion of CNKSR1 high cases had poorly differentiated grade tumors compared to CNKSR1 low cases (22% vs 6%; *p* = 0.04). CNKSR1 expression did not vary appreciably with stage or resection status.

**Table 1:** Characteristics of patients included in the study

		All Patients (*N* = 120)	%
Age group			
	<64	49	41
	65+	71	59
Race			
	White	70	58
	Asian/PI	35	29
	Black or Hispanic	15	13
Gender			
	Male	58	48
	Female	62	52
Grade			
	Well Differentiated (1)	65	54
	Moderately Diff. (2).	34	28
	Poorly Diff. (3).	21	18
Stage			
	Localized	12	10
	Regional	69	58
	Distant	39	33
TNM (N)			
	Lymph Node Negative (N0)	30	25
	Regional Lymph Nodes (N1)	45	38
	Unknown (NX)	45	38
TNM (M)			
	No Metastasis (M0)	77	64
	Distant Metastasis (M1)	39	33
Resection			
	Yes	72	60
	No	48	40
Radiation			
	Yes	30	25
	No	90	75
Location			
	Head	67	56
	Body/Tail	20	17
	Unknown	33	28

**Table 2:** Demographics, tumor characteristics, and treatment by CNKSR1 expression status

		CNKSR1 Low (*N* = 34)	%	CNKSR1 High (*N* = 83)	%	*P*
Age group						0.72
	<64	13	38	36	42	
	65+	21	62	50	58	
Race						0.22
	White	23	68	47	55	
	Asian/PI	6	18	29	34	
	Black or Hispanic	5	15	10	12	
Gender						0.82
	Male	17	50	41	48	
	Female	17	50	45	52	
Grade						0.04
	Well Differentiated (1)	18	53	47	55	
	Moderately Diff. (2).	14	41	20	23	
	Poorly Diff. (3).	2	6	19	22	
Stage						0.86
	Localized	4	12	8	9	
	Regional	20	59	49	57	
	Distant	10	29	29	34	
TNM (N)						0.70
	Lymph Node Negative (N0)	10	29	20	23	
	Regional Lymph Nodes (N1)	11	32	34	40	
	Unknown (NX)	13	38	32	37	
TNM (M)						0.11
	No Metastasis (M0)	21	62	56	65	
	Distant Metastasis (M1)	10	29	29	34	
Resection						0.87
	Yes	20	59	52	60	
	No	14	41	34	40	
Radiation						0.24
	Yes	6	18	24	28	
	No	28	82	62	72	
Location						0.11
	Head	15	44	52	60	
	Body/Tail	5	15	15	17	
	Unknown	14	41	19	22	

CNKSR1 expression status (low = 0,1; high = 2,3 staining intensity) for pancreatic cancer subjects by demographic, tumor characteristic and treatment option variables

**Fig. 6:**
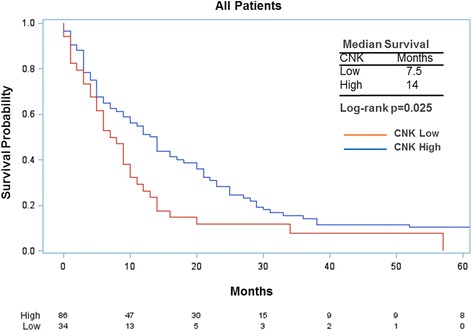
Time from diagnosis to death by CNKSR1 expression (*low* vs *high*)

Figure 6 presents survival probability by CNKSR1 expression. The 86 patients with high CNKSR1 expression survived a median of 14 months from diagnosis compared to 7.5 months for the 34 CNKSR1 low cases (log-rank test, *p* = 0.03).

Table 3 presents the unadjusted and adjusted hazard ratios for pancreatic cancer cases by CNKSR1 expression status. In the unadjusted model, cases with CNKSR1 low tumors had an increased risk of death compared to those with CNKSR1 high tumors with a hazard ratio (HR) of 1.61 (95% CI: 1.06 to 2.46). In a model that adjusted for resection, TNM stage, age, gender, grade, receipt of radiation for localized or regional stage cancer, and race, the hazard ratio for CNKSR1 low compared to CNKSR1 high tumors was equal to 2.146 and the 95% confidence interval ranged from 1.34 to 3.43 (*p* = 0.0014). Using SEER Summary Stage instead of partial TNM Staging revealed a similar hazard ratio (HR) of 1.91 (95% CI: 1.20 to 3.05; data not shown). Other statistically significant variables in the model were, as expected and known from previous clinicopathological multivariate outcome models of pancreas cancer, no resection compared to any resection (HR = 3.78, 95% CI: 2.09 to 6.85), TNM staging indicating regional lymph nodes (HR = 1.89, 95% Cl: 1.21 to 2.97) and distant metastasis (HR = 2.83, 95% Cl: 1.58 to 5.07), and age 65+ (HR = 1.55, 95% CI:1.02 to 2.36). In sensitivity analyses, taking into account missing TNM staging data (N, 49 subjects), the association between low CNKSR1 expression and increased risk of death persisted (data not shown).

**Table 3:** Cox proportional hazard ratio estimates for death in pancreatic cancer subjects

Hazard Ratio Estimates for CNK Negative Status			
Unadjusted model	Hazard Ratio	95% Confidence Limits	*p* value
CNK 0–1	1.611	1.057–2.457	0.027
Adjusted model	Hazard Ratio	95% Confidence Limits	
CNK 0–1	2.146	1.341–3.434	0.0014
No Resection	3.783	2.089–6.852	<0.0001
Distant Metastasis (M0 vs M1)	2.834	1.584–5.069	0.0004
Regional Lymph Nodes (N0 vs N1)	1.893	1.206–2.972	0.0055
65+ Years at Diagnosis	1.550	1.018–2.359	0.041
Male	1.233	0.821–1.851	0.26
Moderately Differentiated	1.014	0.580–1.772	0.96
Poorly Differentiated	0.830	0.508–1.356	0.45
Non-Palliative Radiation^a^	0.674	0.370–1.231	0.19
White	0.611	0.288–1.298	0.20
Asian	0.583	0.268–1.266	0.17

^a^Referent groups for respective variables were subjects with high CNKSR1 expression, those with resections, localized or regional stage cancer at diagnosis, females, cases less than 65 years of age, with localized or regional stage radiation therapy, Black or Hispanic race/ethnicity

### CNKSR1 expression and outcome of patients following surgical resection

**Fig. 7:**
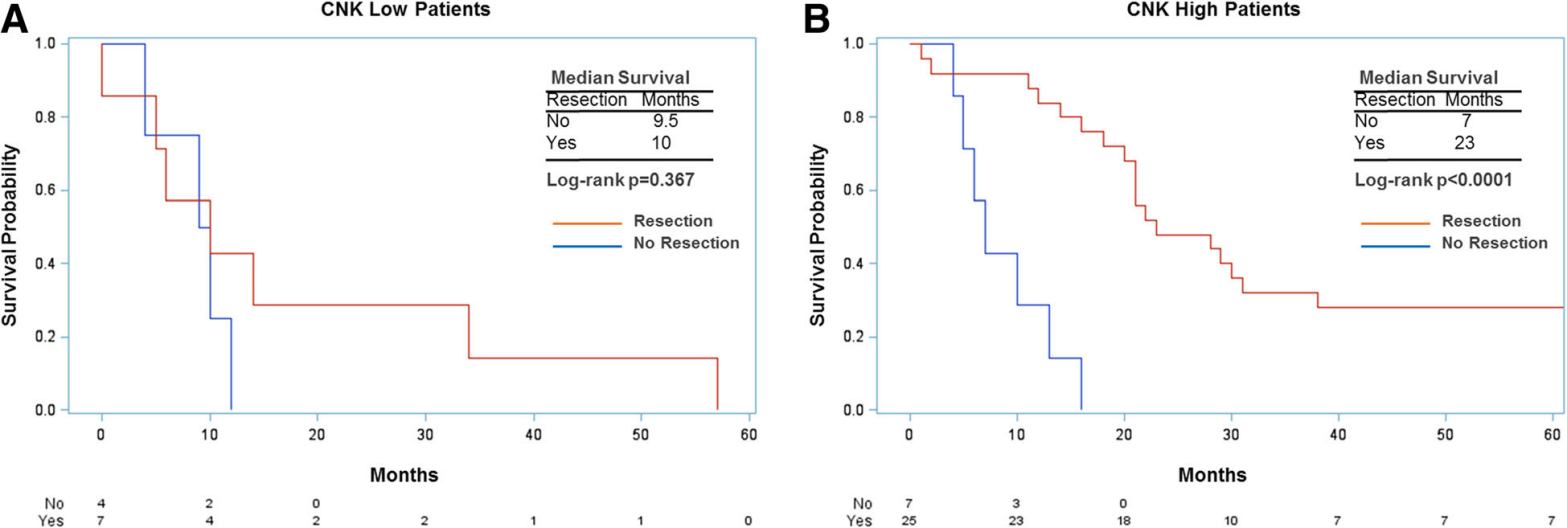
Survival stratified by resection and CNKSR1 expression status. **a** Survival in limited subset of primary tumor biopsy specimens showing low CNKSR1 expression by resection status (*p* = 0.367). **b** Survival in patients with high CNKSR1 expression by resection status (*p* < 0.0001)

Since the unfavorable course of low CNKSR1 expressing tumors suggests a more aggressive tumor biology, we investigated the possibility that the impact of resection on survival was diminished in this group. This analysis was limited to primary, non-metastatic tumor biopsies, since this represents the group where CNKSR1 status might be used in pre-operative decision-making. Patients with CNKSR1 low expression did not show an associated survival difference between resected patients and non-resected cases (*p* = 0.3666, Fig. 7), while patients with high CNKSR1 expression did have a positive association between resection status and survival (*p* < 0.0001). Thus, CNKSR1 expression status might capture unfavorable tumor biology for surgical resection for pancreatic cancer, and may aid in pre-operative treatment selection.

### Phospho-ERK expression in relation to CNKSR1 expression pattern in the SEER pancreatic cancer TMA

**Fig. 8:**
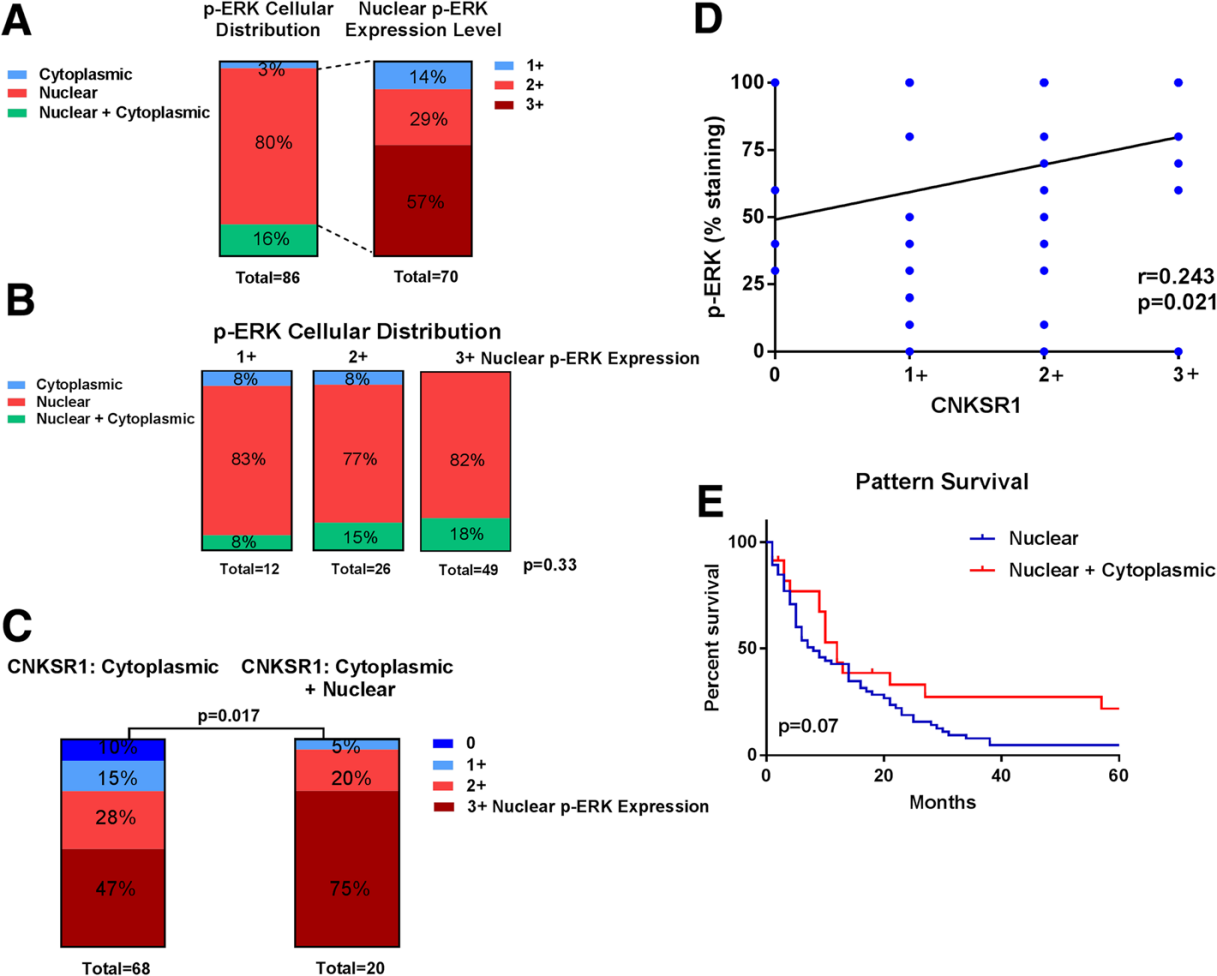
Cellular distribution of CNKSR1 is associated with p-ERK expression levels in pancreatic cancer specimens. **a** p-ERK cellular distribution and nuclear expression levels (scored as 1+, 2+, and 3+) in 86 cases of the SEER Pancreatic Cancer TMA. **b** Nuclear expression levels of p-ERK (scored as 1+, 2+, and 3+) are not associated with presence of cytoplasmic p-ERK expression pattern. **c** Pancreatic cancer specimens with concomitant cytoplasmic and nuclear CNKSR1 expression have increased nuclear p-ERK staining (scored by 0, 1+, 2+, and 3+ intensity levels) compared to pancreatic cancers with cytoplasmic CNKSR1 staining only (Mann Whitney U test; 2-tailed). **d** Cytoplasmic CNKSR1 expression levels (scored semiquantitatively as 0, 1+, 2+, and 3+) and nuclear p-ERK expression levels (scored as % positive cells) (Pearson’s correlation coefficient test; 2-tailed). **e** Kaplan-Meier survival analysis of pancreatic cancer cases from SEER Pancreatic Cancer TMA with p-ERK stratified by cellular distribution of p-ERK (nuclear vs nuclear and cytoplasmic) (log-rank; 2-tailed)

To gain more insight into the mechanism of action and role of CNKSR1 in MAPK pathway regulation, we next measured expression levels of phospho-ERK in 86 matched cases of the SEER Pancreatic Cancer TMA previously evaluated and scored for CNKSR1 expression levels. As phosphorylation of the Extracellular-signal Regulated Kinase (ERK) induces nuclear translocation, p-ERK expression pattern was found to be predominantly nuclear, with only 14 cases (16%) showing additional cytoplasmic staining and three cases (3%) showing exclusive cytoplasmic staining without nuclear expression (Fig. 8a). There was significant heterogeneity in the degree of nuclear p-ERK levels, with 12 cases (14%) staining weak (1+), 26 cases (29%) moderate (2+), and 49 cases (57%) strong (3+). The presence of concomitant cytoplasmic staining was not associated with nuclear pERK1/2 expression levels (Fig. 8b). We next examined whether the cellular distribution of CNKSR1 was correlated with p-ERK expression levels scored by intensity of p-ERK immunostaining. Figure 8C shows nuclear p-ERK expression levels (0, 1+, 2+, 3+)) by CNKSR1 cellular distribution (cytoplasmic CNKSR1 expression only vs cytoplasmic and nuclear) in pancreas cancer specimens of the SEER Pancreatic Cancer TMA (Mann Whitney U test; *p* = 0.017). To test whether expression levels of the two proteins are correlated as well, including if a possible negative correlation between cytoplasmic CNKSR1 expression levels (0, 1+, 2+, and 3+) and nuclear p-ERK levels (in % of tumor cells with nuclear staining) exists, Pearson’s correlation coefficient testing was employed (Fig. 8d). There was no correlation between cytoplasmic CNKSR1 expression levels and nuclear p-ERK expression levels (*r* = 0.24; *p* < 0.05). There was a trend towards different overall outcome by stratification of patients by cellular distribution of p-ERK expression (nuclear vs nuclear and cytoplasmatic) (log rank test; HR = 1.61; *p* = 0.07). These finding suggest that cellular distribution of CNKSR1, rather than expression levels, might be involved in MAPK pathway and p-ERK regulation.

## Discussion

In this study, we examined the expression levels of the scaffolding kinase CNKSR1 in pancreatic cancer surgical resection specimens and evaluated their impact on clinical outcome, including overall survival (OS). We found, in a limited set of matched normal and tumor specimens, that CNKSR1 expression is elevated in cancer tissue compared to normal uninvolved pancreas tissue. Elevated CNKSR1 expression correlated with improved survival as an independent prognostic marker using multivariate analysis, with patients in the low CNKSR1 expression group having a median OS that is nearly half that of patients with high CNKSR1 expression. In addition, we attempted to determine whether CNKSR1 status might affect the survival difference associated with resection in pancreatic cancer patients. If validated in a larger patient sample, such information might be useful in pre-operative decision-making.

CNKSR1 expression has not been previously shown to be associated with survival. This is in contrast to KSR1, the other major scaffolding protein of the MAPK/ERK pathway. KSR1, which tightly regulates MAPK/ERK signaling output, has been shown to be overexpressed in endometrial and colon carcinoma and high expression levels reported to be associated with decreased survival in breast cancer [14, 15, 27]. KSR1 has been shown to impact tumor biology, including in preclinical models of pancreas cancer, as well as response to chemotherapy [12]. To date, such information is largely lacking for CNKSR1, a binding partner and regulator of KSR1 and the MAPK pathway.

One of the main functions of the scaffold protein CNKSR1 is the regulation of other cancer-related signaling pathways integrating output to different intracellular signaling cascades upon cellular stimulation by extracellular cues [11, 12, 17–20]. In breast and cervical cancer cell models, a pro-oncogenic potential has been demonstrated through ERK-independent AKT-FoxO and NF-kappaB pathways [17, 19]. In a colorectal cancer cell model, CNKSR1 has been shown to facilitate pro-apoptotic signaling via RASSF1A [18]. Importantly, a recent cell-based study expressing mutants of known phosphorylation sites of CNKSR1 in cancer cells identified sites linked to nuclear translocation with concomitant activation of MAPK pathway-driven serum response element (SRE) gene expression providing, for the first time, a direct correlation of cellular CNKSR1 distribution and MAPK pathway signaling output. [21] These findings are in line with the identified correlation of CNKSR1 cellular distribution (cytoplasmic only vs cytoplasmic and nuclear) and nuclear p-ERK expression levels observed in our study. The finding that pancreatic tumors which have nuclear CNKSR1 expression in addition to the predominant cytoplasmic expression of CNKSR1 show higher nuclear p-ERK expression levels suggests that cellular localization, rather than absolute CNKSR1 expression levels, might be one of the mechanisms of CNKRS1-mediated control of MAPK pathway activity. In this regard, Fisher and colleagues showed in an elegant study with a set of different phosphomimetic mutant constructs that phosphorylation of Tyr 519 recruits CNKSR1 to the nucleus and phosphorylation of Tyr 26 enables CNKSR1 to activate MAPK pathway-governed serum response element (SRE) gene transcription [21]. Previous smaller correlative biomarker studies did show that increased nuclear p-ERK staining levels were associated with poorer survival in pancreas cancer [28, 29]. Such MAPK pathway regulation by nuclear translocation possibly induced by posttranslational modifications including phosphorylation of select sites of CNKSR1, appears in line with our findings of a correlation between the cellular distribution of CNKSR1 and nuclear p-ERK expression levels [30].

While there have been more than 100 correlative biomarkers described investigating pancreatic cancer, there have been less than a dozen studies which correlated putative markers with survival outcome [31]. Similar to the hazard ratio (HR) estimates for death reported for CNKSR1 in this study, the majority of previously described prognostic biomarker studies reported HRs for death ranging from 1.5 to 4 [31]. Exceptions to these studies are, among others, the recently reported study of 13 putative pancreas cancer biomarkers from investigators at the Memorial Sloan-Kettering Cancer Center (MSKCC) describing two biomarkers with HRs exceeding ten [4]. It is also noteworthy that of the biomarkers reported to be associated with survival (reviewed by Winter et al. 2013), there are a number of proteins either indirectly or directly connected to MAPK/ERK signaling (Ras, ERBB2, Myc) [4, 31].

A limitation of this study was that the dataset did not include all clinical variables, such as complete TNM staging and chemotherapy treatment, and thus we could not evaluate all variables that might impact survival. While the HRs of known clinical (TNM system N and M stage) and pathological variables in the Cox proportional hazard ratio model yielded previously known associations with outcome, the T variable of the AJCC TNM staging system was not fully collected and only incompletely captured by the stage variable (localized vs regional). While this somehow limits direct comparability to previous studies, prospectively evaluated and validated nomograms on surgical outcome of pancreas cancer have shown that, by far, M and N stage, as captured in our study, are the strongest predictors of outcome [32, 33]. In addition, data regarding systemic therapy was not available, though nearly all cases were diagnosed prior to the routine use of FOLFIRINOX or Gemcitabine/nab-Paclitaxel.

We consider validation of the CNKSR1 expression pattern used for the correlative outcome studies in the two independent cohorts of pancreas cancer surgical specimens a strength of the study. Co-staining of matched SEER Pancreatic Cancer TMA specimens with p-ERK suggests a correlation between cellular distribution of CNKSR1 and nuclear p-ERK expression levels, possibly indicating regulation of MAPK pathway signaling less connected to absolute CNKSR1 expression levels. In addition, the SEER TMA studied has been used in multiple previous biomarker studies, several of which have been validated in additional larger cohorts [4, 22]. CNKSR1 may represent an additional prognostic marker in pancreatic cancer, and further validation in a larger cohort of recent patients is needed.

## Conclusion

In this correlative tissue study including independent pancreatic cancer tissue microarrays from different sources, CNKSR1 expression was found to be an independent marker of patient outcome. We identified CNKSR1 low patients at high risk for disease progression suggesting that there might be distinct differences in tumor biology between CNKSR1 high and CNKRS1 low tumors. In CNKSR1 low expressing tumors, patients did not show any difference in survival when stratified by resection status whereas patients with high CNKSR1 expression levels who underwent resection had significantly improved outcome compared to non-resected patients in this group. Combination of CNKSR1 expression levels with current clinicopathological prognostic features might improve risk stratification and treatment selection.

## Abbreviations

CNKSR1: Connector enhancer of the kinase suppressor of ras-1
HR: Hazard ratio
KSR1: Kinase suppressor of ras-1
PDAC: Pancreatic ductal adenocarcinoma
SEER: Surveillance, epidemiology, and end results
TMA: Tissue microarray
